# Remedies for Post-Partum Hemorrhage: Secale

**Published:** 1888-04

**Authors:** J. D. Tyrrell


### REMEDIES FOR POST-PARTUM HEMORRHAGE.

### SECALE.

Mr. President and Gentlemen: Should it ever be our province to stand face to face with that appalling catastrophe, post-partum hemorrhage; to feel the awful presence of the Angel of Death, as the faithful wife and devoted mother gives birth to the new life, only to find her own ebbing away in a crimson tide as die the waves upon the shore, it may be ere she can imprint upon her baby's lips the holy kiss of a mother's love; should such be our province, we can praise a beneficent Creator for raising up the illustrious Samuel Hahnemann to proclaim the law, and promulgate the doctrine of "*Similia similibus curantur.*" By being *faithful* priests in the temple of Similia we can confidently say to the dread visitor, "Hitherto shalt thou come, but no further," and, administering the chosen remedy, see the crimson flood subside, the gray shadow recede even as "the sun returned ten degrees," and the mother come back from the "valley of the shadow of death."

I will now direct your attention for a little space to some of the characteristic features (compared with others) of a friend that will stand by us in this our hour of dire extremity; by such a study we may surely recognize the friend in the *faithful* likeness portrayed in the symptoms of the sick.

Secale seems to prefer old and decrepit persons; women who are thin and scrawny; lax muscular system. Face usually pale and wan, it *may* be dark red, eyes sunken; face pinched with coldness. Respiration may become anxious, panting, sighing, sobbing, or unequal; pulsation of heart intermits. There is burning of skin all over as from sparks, ameliorated by cold

(contra Ars.). The patient often complains of external coldness and *sensation* of coldness, but she cannot bear heat or being covered up. Tingling and numbness of limbs and formication under skin are quite characteristic of Secale; skin is flabby; cold and dry; wrinkled skin; cold, clammy sweat all over body, especially the limbs.

The hemorrhage of Secale is usually painless; passive (Ustilago); and worse from the slightest motion; here it resembles Calc., Crocus, and Sabina, in the last however, though worse from slightest motion, the hemorrhage is very much relieved by walking.

The *appearance* of the blood is quite worthy of notice, and if you will bear with me, I will *compare* a few similars: In Secale, the blood is dark red (Cham., Sabina); black, liquid, viscous, and of bad odor. Similarly Crocus has *black* flow, only it is *stringy* and viscid; Kreos. has blood black, flows in a stream; Platina has *hard and black clots* mixed with fluid blood, which is dark and tarry; body feels as if growing larger.

Secale pulse is usually small, weak, and thread-like in hemorrhages. By the way, let me call your attention to the fact that Secale *may* have blood of a light color, in which case it is *bright red*, generally coagulated, flows *intermittingly* (Ipecac., *continuous* stream, *bright red blood*); with pain; *pulse hard and quick.* Ustilago has bright red blood, when the clots are large, bearing down as if everything would come through, like Bell., which has involuntary groans, which relieve. In Secale the os uteri does not contract, is painful to touch, and feels hard (Ustilago, os and cervix are soft and spongy).

To sum up, Secale has dissolution of blood cells; relaxation of vessels and oozing from capillaries; *thin, dark, and black blood.* General aggravation from being touched; from warm applications and external warmth (pain in uterus and limbs).

*All symptoms and conditions* are relieved by being uncovered and by cold (contra Ars.). After Secale has controlled the hemorrhage, you *may* find your patient does not progress beyond a given point; does not gain in strength, and still seems anæmic; in such a condition you will most likly find China the best complementary remedy. J. D. TYRRELL.

